# 2-[(2-Chloro­phen­yl)imino­meth­yl]-4,6-di­iodo­phenol

**DOI:** 10.1107/S1600536812007325

**Published:** 2012-02-24

**Authors:** Hao Ji, Yong-An Yang, Hua-Ping Ma, Hai-Liang Zhu

**Affiliations:** a State Key Laboratory of Pharmaceutical Biotechnology, Nanjing University, Nanjing 210093, People’s Republic of China, and Jiangsu Tiansheng Pharmaceutical Company Limited, Jurong Jiangsu 212415, People’s Republic of China

## Abstract

The asymmetric unit of the title compound, C_13_H_8_ClI_2_NO, contains half of the mol­ecule situated on a mirror plane. The hy­droxy group is involved in the formation of an intra­molecular O—H⋯N hydrogen bond. π–π inter­actions between the benzene rings of neighbouring mol­ecules [centroid–centroid distance = 3.629 (3) Å] form stacks along the *b* axis. In the crystal, weak C—H⋯O and C—H⋯Cl inter­actions are observed.

## Related literature

For standard bond distances, see: Allen *et al.* (1987 ▶). For the crystal structures of related compounds, see: Francis *et al.* (2003 ▶); Weiser *et al.* (2006 ▶); Barba *et al.* (2009 ▶).
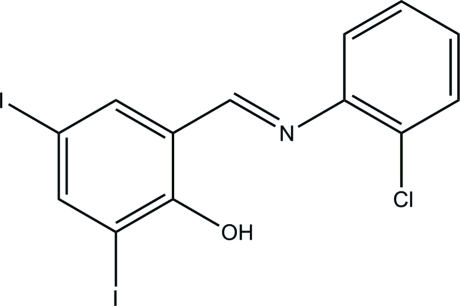


## Experimental

### 

#### Crystal data

C_13_H_8_ClI_2_NO
*M* *_r_* = 483.45
Orthorhombic, 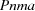

*a* = 15.8432 (17) Å
*b* = 6.9942 (8) Å
*c* = 13.1975 (14) Å
*V* = 1462.4 (3) Å^3^
*Z* = 4
Mo *K*α radiation
μ = 4.47 mm^−1^
*T* = 296 K
0.20 × 0.10 × 0.10 mm

#### Data collection

Bruker SMART CCD area-detector diffractometer
Absorption correction: multi-scan (*SADABS*; Sheldrick, 1996 ▶) *T*_min_ = 0.468, *T*_max_ = 0.663
9861 measured reflections
1829 independent reflections
1659 reflections with *I* > 2σ(*I*)
*R*_int_ = 0.029

#### Refinement

*R*[*F* ^2^ > 2σ(*F* ^2^)] = 0.039
*wR*(*F* ^2^) = 0.144
*S* = 0.98
1829 reflections
113 parameters
1 restraint
H atoms treated by a mixture of independent and constrained refinement
Δρ_max_ = 1.13 e Å^−3^
Δρ_min_ = −0.76 e Å^−3^

### 

Data collection: *SMART* (Bruker, 1998 ▶); cell refinement: *SAINT* (Bruker, 1998 ▶); data reduction: *SAINT*; program(s) used to solve structure: *SHELXS97* (Sheldrick, 2008 ▶); program(s) used to refine structure: *SHELXL97* (Sheldrick, 2008 ▶); molecular graphics: *SHELXTL* (Sheldrick, 2008 ▶); software used to prepare material for publication: *SHELXTL*.

## Supplementary Material

Crystal structure: contains datablock(s) global, I. DOI: 10.1107/S1600536812007325/cv5247sup1.cif

Structure factors: contains datablock(s) I. DOI: 10.1107/S1600536812007325/cv5247Isup2.hkl

Supplementary material file. DOI: 10.1107/S1600536812007325/cv5247Isup3.cml

Additional supplementary materials:  crystallographic information; 3D view; checkCIF report

## Figures and Tables

**Table 1:** Hydrogen-bond geometry (Å, °)

*D*—H⋯*A*	*D*—H	H⋯*A*	*D*⋯*A*	*D*—H⋯*A*
O1—H1*A*⋯N1	0.84 (2)	1.95 (8)	2.568 (8)	130 (9)
C11—H11*A*⋯O1^i^	0.93	2.57	3.496 (8)	178
C12—H12*A*⋯Cl1^i^	0.93	2.83	3.640 (8)	147

Symmetry code: (i) 
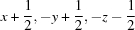
.

**Fig. 1.**
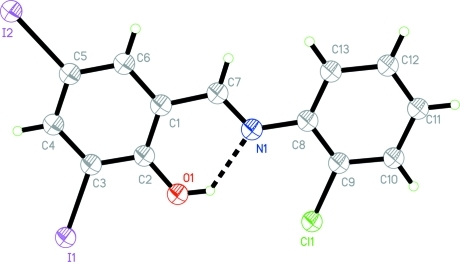
The molecular structure of the title compound, showing the atom labelling scheme. The displacement ellipsoids are drawn at the 30% probability level. Intramolecular hydrogen bond is indicated by a dashed line.

## supplementary crystallographic information

### Comment

Schiff bases have been extensively studied for their structures and applications. In the present paper, we present the title compound (I) (Fig. 1) - a new Schiff base compound.

The asymmetric unit of (I) contains a half of the molecule situated on a mirror plane. The molecule of the compound adopts an *E* configuration with respect to the C=N bond. The hydroxy group is involved in formation of intramolecular O—H···N hydrogen bond (Table 1). Bond distances are within normal values (Allen *et al.*, 1987), and are comparable with those reported in the literature for related compounds (Weiser *et al.*, 2006; Barba *et al.*, 2009; Francis *et al.*, 2003).

π–π Interactions between the benzene rings of the neighbouring molecules [centroid-centroid distance = 3.629 (3) Å] form stacks along axis *b*. Weak intermolecular C—H···O and C—H···Cl interactions (Table 1) consolidate further the crystal packing.

### Experimental

3,5-Diiodosalicylaldehyde (0.37 g, 1 mmol) and 2-chlorophenylamine (0.13 g, 1 mmol) were mixed in ethanol (20 ml). The mixture was stirred at room temperature for 30 min to give a yellow solution. Yellow block-shaped single crystals were obtained by slow evaporation of the solution containing the compound in air.

### Refinement

C-bound H atoms were positioned geometrically and allowed to ride on their parent atoms, with C—H = 0.93 Å and U_iso_ = 1.2 U_eq_(C). Atom H1 was located on a difference map and isotropically refined.

### Crystal data

**Table:**

C_13_H_8_ClI_2_NO	*D*_x_ = 2.196 Mg m^−^^3^
*M**_r_* = 483.45	Mo *K*α radiation, λ = 0.71073 Å
Orthorhombic, *P**n**m**a*	Cell parameters from 897 reflections
*a* = 15.8432 (17) Å	θ = 2.4–24.5°
*b* = 6.9942 (8) Å	µ = 4.47 mm^−^^1^
*c* = 13.1975 (14) Å	*T* = 296 K
*V* = 1462.4 (3) Å^3^	Block, yellow
*Z* = 4	0.20 × 0.10 × 0.10 mm
*F*(000) = 896	

### Data collection

**Table:**

Bruker SMART CCD area-detector diffractometer	1829 independent reflections
Radiation source: fine-focus sealed tube	1659 reflections with *I* > 2σ(*I*)
Graphite monochromator	*R*_int_ = 0.029
ω scans	θ_max_ = 27.6°, θ_min_ = 2.0°
Absorption correction: multi-scan (*SADABS*; Sheldrick, 1996)	*h* = −20→20
*T*_min_ = 0.468, *T*_max_ = 0.663	*k* = −9→9
9861 measured reflections	*l* = −17→17

### Refinement

**Table:**

Refinement on *F*^2^	Secondary atom site location: difference Fourier map
Least-squares matrix: full	Hydrogen site location: inferred from neighbouring sites
*R*[*F*^2^ > 2σ(*F*^2^)] = 0.039	H atoms treated by a mixture of independent and constrained refinement
*wR*(*F*^2^) = 0.144	*w* = 1/[σ^2^(*F*_o_^2^) + (0.1*P*)^2^ + 4.5*P*] where *P* = (*F*_o_^2^ + 2*F*_c_^2^)/3
*S* = 0.98	(Δ/σ)_max_ < 0.001
1829 reflections	Δρ_max_ = 1.13 e Å^−^^3^
113 parameters	Δρ_min_ = −0.76 e Å^−^^3^
1 restraint	Extinction correction: *SHELXL97* (Sheldrick, 2008), Fc^*^=kFc[1+0.001xFc^2^λ^3^/sin(2θ)]^-1/4^
Primary atom site location: structure-invariant direct methods	Extinction coefficient: 0.0030 (6)

### Special details

**Table:**

Geometry. All e.s.d.'s (except the e.s.d. in the dihedral angle between two l.s. planes) are estimated using the full covariance matrix. The cell e.s.d.'s are taken into account individually in the estimation of e.s.d.'s in distances, angles and torsion angles; correlations between e.s.d.'s in cell parameters are only used when they are defined by crystal symmetry. An approximate (isotropic) treatment of cell e.s.d.'s is used for estimating e.s.d.'s involving l.s. planes.
Refinement. Refinement of *F*^2^ against ALL reflections. The weighted *R*-factor *wR* and goodness of fit *S* are based on *F*^2^, conventional *R*-factors *R* are based on *F*, with *F* set to zero for negative *F*^2^. The threshold expression of *F*^2^ > σ(*F*^2^) is used only for calculating *R*-factors(gt) *etc*. and is not relevant to the choice of reflections for refinement. *R*-factors based on *F*^2^ are statistically about twice as large as those based on *F*, and *R*- factors based on ALL data will be even larger.

### Fractional atomic coordinates and isotropic or equivalent isotropic displacement parameters (Å^2^)

**Table:**

	*x*	*y*	*z*	*U*_iso_*/*U*_eq_	
I1	0.19354 (3)	0.2500	0.09648 (5)	0.0692 (3)	
I2	0.44288 (3)	0.2500	0.43751 (4)	0.0582 (3)	
C3	0.3198 (4)	0.2500	0.1448 (5)	0.0444 (16)	
C2	0.3847 (4)	0.2500	0.0744 (6)	0.0470 (17)	
C5	0.4191 (4)	0.2500	0.2817 (5)	0.0403 (14)	
O1	0.3668 (3)	0.2500	−0.0237 (4)	0.076 (2)	
C4	0.3362 (4)	0.2500	0.2491 (5)	0.0395 (13)	
H4A	0.2921	0.2500	0.2956	0.047*	
C6	0.4851 (4)	0.2500	0.2125 (5)	0.0404 (14)	
H6A	0.5406	0.2500	0.2352	0.048*	
Cl1	0.46567 (14)	0.2500	−0.26597 (17)	0.0654 (6)	
C10	0.6308 (6)	0.2500	−0.3086 (7)	0.056 (2)	
H10A	0.6146	0.2500	−0.3764	0.068*	
N1	0.5265 (4)	0.2500	−0.0572 (4)	0.0456 (14)	
C12	0.7400 (5)	0.2500	−0.1822 (9)	0.073 (3)	
H12A	0.7968	0.2500	−0.1647	0.088*	
C13	0.6773 (5)	0.2500	−0.1049 (8)	0.065 (2)	
H13A	0.6932	0.2500	−0.0370	0.078*	
C8	0.5925 (4)	0.2500	−0.1305 (6)	0.0437 (15)	
C1	0.4679 (4)	0.2500	0.1090 (5)	0.0353 (13)	
C9	0.5697 (4)	0.2500	−0.2316 (6)	0.0442 (15)	
C11	0.7168 (7)	0.2500	−0.2820 (9)	0.078 (3)	
H11A	0.7579	0.2500	−0.3323	0.094*	
C7	0.5388 (4)	0.2500	0.0385 (6)	0.0432 (15)	
H7A	0.5937	0.2500	0.0635	0.052*	
H1A	0.404 (5)	0.2500	−0.069 (6)	0.06 (3)*	

### Atomic displacement parameters (Å^2^)

**Table:**

	*U*^11^	*U*^22^	*U*^33^	*U*^12^	*U*^13^	*U*^23^
I1	0.0239 (3)	0.1386 (7)	0.0450 (4)	0.000	−0.00480 (18)	0.000
I2	0.0463 (3)	0.0979 (5)	0.0303 (3)	0.000	−0.00509 (18)	0.000
C3	0.025 (3)	0.078 (5)	0.030 (3)	0.000	−0.005 (2)	0.000
C2	0.024 (3)	0.079 (5)	0.037 (4)	0.000	−0.006 (3)	0.000
C5	0.028 (3)	0.065 (4)	0.027 (3)	0.000	−0.004 (2)	0.000
O1	0.027 (3)	0.171 (7)	0.029 (3)	0.000	−0.002 (2)	0.000
C4	0.032 (3)	0.055 (4)	0.031 (3)	0.000	0.001 (2)	0.000
C6	0.025 (3)	0.054 (4)	0.043 (4)	0.000	−0.006 (2)	0.000
Cl1	0.0433 (10)	0.1094 (18)	0.0435 (11)	0.000	−0.0023 (8)	0.000
C10	0.057 (5)	0.067 (5)	0.046 (4)	0.000	0.024 (4)	0.000
N1	0.032 (3)	0.070 (4)	0.035 (3)	0.000	0.008 (2)	0.000
C12	0.018 (3)	0.122 (8)	0.081 (7)	0.000	0.010 (4)	0.000
C13	0.028 (3)	0.107 (7)	0.060 (6)	0.000	0.004 (3)	0.000
C8	0.033 (3)	0.058 (4)	0.041 (4)	0.000	0.011 (3)	0.000
C1	0.023 (3)	0.049 (3)	0.034 (3)	0.000	−0.002 (2)	0.000
C9	0.033 (3)	0.060 (4)	0.039 (4)	0.000	0.008 (3)	0.000
C11	0.070 (6)	0.091 (7)	0.075 (7)	0.000	0.050 (6)	0.000
C7	0.023 (3)	0.061 (4)	0.046 (4)	0.000	0.003 (3)	0.000

### Geometric parameters (Å, º)

**Table:**

I1—C3	2.100 (6)	C10—C11	1.407 (15)
I2—C5	2.090 (6)	C10—H10A	0.9300
C3—C2	1.386 (10)	N1—C7	1.278 (10)
C3—C4	1.400 (9)	N1—C8	1.425 (8)
C2—O1	1.325 (9)	C12—C11	1.368 (16)
C2—C1	1.395 (9)	C12—C13	1.424 (13)
C5—C4	1.383 (9)	C12—H12A	0.9300
C5—C6	1.388 (9)	C13—C8	1.385 (11)
O1—H1A	0.84 (2)	C13—H13A	0.9300
C4—H4A	0.9300	C8—C9	1.382 (11)
C6—C1	1.393 (9)	C1—C7	1.459 (9)
C6—H6A	0.9300	C11—H11A	0.9300
Cl1—C9	1.709 (7)	C7—H7A	0.9300
C10—C9	1.404 (9)		
			
C2—C3—C4	121.4 (6)	C11—C12—H12A	119.9
C2—C3—I1	120.2 (5)	C13—C12—H12A	119.9
C4—C3—I1	118.4 (5)	C8—C13—C12	120.1 (9)
O1—C2—C3	119.8 (6)	C8—C13—H13A	120.0
O1—C2—C1	121.5 (6)	C12—C13—H13A	120.0
C3—C2—C1	118.8 (6)	C9—C8—C13	119.3 (7)
C4—C5—C6	120.7 (6)	C9—C8—N1	117.6 (6)
C4—C5—I2	118.5 (5)	C13—C8—N1	123.0 (7)
C6—C5—I2	120.8 (5)	C6—C1—C2	120.4 (6)
C2—O1—H1A	123 (7)	C6—C1—C7	118.4 (6)
C5—C4—C3	118.9 (6)	C2—C1—C7	121.3 (6)
C5—C4—H4A	120.6	C8—C9—C10	121.2 (7)
C3—C4—H4A	120.6	C8—C9—Cl1	120.6 (5)
C5—C6—C1	119.9 (6)	C10—C9—Cl1	118.2 (7)
C5—C6—H6A	120.0	C12—C11—C10	120.0 (8)
C1—C6—H6A	120.0	C12—C11—H11A	120.0
C9—C10—C11	119.1 (8)	C10—C11—H11A	120.0
C9—C10—H10A	120.4	N1—C7—C1	120.8 (6)
C11—C10—H10A	120.4	N1—C7—H7A	119.6
C7—N1—C8	124.0 (6)	C1—C7—H7A	119.6
C11—C12—C13	120.2 (8)		

### Hydrogen-bond geometry (Å, º)

**Table:**

*D*—H···*A*	*D*—H	H···*A*	*D*···*A*	*D*—H···*A*
O1—H1*A*···N1	0.84 (2)	1.95 (8)	2.568 (8)	130 (9)
C11—H11*A*···O1^i^	0.93	2.57	3.496 (8)	178
C12—H12*A*···Cl1^i^	0.93	2.83	3.640 (8)	147

Symmetry code: (i) *x*+1/2, −*y*+1/2, −*z*−1/2.

## References

[bb1] Allen, F. H., Kennard, O., Watson, D. G., Brammer, L., Orpen, A. G. & Taylor, R. (1987). *J. Chem. Soc. Perkin Trans. 2*, pp. S1–19.

[bb2] Barba, V., Hernandez, R., Hopfl, H., Santillan, R. & Farfan, N. (2009). *J. Organomet. Chem.* **694**, 2127–2133.

[bb3] Bruker (1998). *SMART* and *SAINT* Bruker AXS Inc., Madison, Wisconsin, USA.

[bb4] Francis, S., Mu­thiah, P. T., Venkatachalam, G. & Ramesh, R. (2003). *Acta Cryst.* E**59**, o1045–o1047.

[bb5] Sheldrick, G. M. (1996). *SADABS* University of Göttingen, Germany.

[bb6] Sheldrick, G. M. (2008). *Acta Cryst.* A**64**, 112–122. 10.1107/S0108767307043930 18156677

[bb7] Weiser, M.-S., Wesolek, M. & Mulhaupt, R. (2006). *J. Organomet. Chem.* **691**, 2945–2952.

